# A Chymase Inhibitory RNA Aptamer Improves Cardiac Function and Survival after Myocardial Infarction

**DOI:** 10.1016/j.omtn.2018.11.001

**Published:** 2018-11-13

**Authors:** Denan Jin, Shinji Takai, Yosuke Nonaka, Satoko Yamazaki, Masatoshi Fujiwara, Yoshikazu Nakamura

**Affiliations:** 1 Department of Innovative Medicine, Graduate School of Medicine, Osaka Medical College, 2-7 Daigaku-machi, Takatsuki, Osaka 569-8686, Japan; 2 RIBOMIC Inc., Minato-ku, Tokyo 108-0071, Japan; 3 Institute of Medical Science, The University of Tokyo, Minato-ku, Tokyo 108-8639, Japan

**Keywords:** chymase inhibitor, RNA aptamer, myocardial infarction, cardiac function, survival

## Abstract

We have reported that mast cell chymase, an angiotensin II-generating enzyme, is important in cardiovascular tissues. Recently, we developed a new chymase-specific inhibitory RNA aptamer, HA28, and we evaluated the effects of HA28 on cardiac function and the mortality rate after myocardial infarction. Echocardiographic parameters, such as the left ventricular ejection fraction, fractional shortening, and the ratio of early to late ventricular filling velocities, were significantly improved by treatment with HA28 after myocardial infarction. The mortality rate was significantly reduced in the HA28-treated group. Cardiac chymase activity and chymase gene expression were significantly higher in the vehicle-treated myocardial infarction group, and these were markedly suppressed in the HA28-treated myocardial infarction group. The present study provides the first evidence that a single-stranded RNA aptamer that is a chymase-specific inhibitor is very effective in the treatment of acute heart failure caused by myocardial infarction. Chymase may be a new therapeutic target in post-myocardial infarction pathophysiology.

## Introduction

Myocardial infarction (MI) is caused by sudden occlusion of the coronary arteries, and the mortality rate following MI is rather high. After MI, patients may survive but most will develop heart failure (HF).^1^ The poor prognosis after MI may be due to the following reasons. First, acute occlusion of large coronary arteries damages the heart muscle, and it not only elicits a significant decrease in cardiac output (CO) but also an increase in the occurrence of lethal arrhythmias resulting from an imbalance in cardiac electrical conduction. Ventricular fibrillation is the most important cause of sudden death following MI.^2^, ^3^ Second, human adult cardiomyocytes, which are in an advanced stage of terminal differentiation, can no longer proliferate, and, therefore, these cells cannot regenerate once they are lost in the heart after MI.

To compensate for the decreased cardiac function due to massive necrosis of cardiomyocytes, several neurohumoral systems are activated. For example, in response to the decrease in CO after MI, acute arterial baroreflex-mediated activation of the sympathetic nervous system is provoked. An increase in catecholamines, such as noradrenaline and adrenaline, through the stimulation of cardiac β1 adrenergic receptors present in the non-infarcted myocardium increases cardiac contractibility, and the reduced CO may be partially restored during the acute phase of MI.^4^ On the other hand, the increase in circulating adrenaline also stimulates renal β1 adrenergic receptors to secrete renin, which is the rate-limiting enzyme in the renin-angiotensin system (RAS), and, as a result, circulating angiotensin II (Ang II) and aldosterone are increased.^5^ Aldosterone, through the promotion of Na^+^ resorption in the kidney, increases the blood volume, and post-MI CO may improve.^6^

These acute compensatory mechanisms are beneficial for rescuing patients from acute HF after MI. However, sustained activation of the sympathetic nervous system and RAS may have harmful effects on post-MI pathophysiology. Both adrenaline and Ang II are powerful vasoconstrictors, and increases in these factors can lead to chronic deterioration of post-MI cardiac function caused by the cardiac afterload increase. The increase in blood volume by aldosterone also increases the cardiac preload and worsens post-MI symptoms.^7^ In addition, chronic activation of RAS may result in abnormal cardiac remodeling, such as cardiac hypertrophy and fibrosis. These organic changes may cause post-MI HF to be more severe and irreversible.^8^, ^9^ Therefore, pharmacological intervention to counteract RAS activation may be beneficial after MI.

Recently, chymase has been shown to play important roles in the regulation of local Ang II formation in human cardiovascular tissues. Chymase is a chymotrypsin-like serine protease, which is mainly contained in the secretory granules of mast cells. Like angiotensin-converting enzyme, chymase can also cleave Ang I to Ang II when mast cells undergo degranulation.^10^, ^11^ Importantly, in the human heart, chymase accounts for about 80%–90% of Ang II formation,^12^ suggesting the importance of cardiac chymase in Ang II-related cardiac diseases. On the other hand, chymase can also enzymatically cleave pro-matrix metalloproteinase (MMP)9 and latent transforming growth factor (TGF)-β1 to their active forms.^13^, ^14^, ^15^ These two factors participate closely in the cardiac remodeling that happens following MI. Therefore, the effects of chymase on post-MI pathophysiology may extend beyond an effect on Ang II generation.

In this study, the effects of chymase inhibition by a newly synthesized RNA aptamer against hamster chymase were examined in a hamster left coronary artery (LCA) ligation model. An aptamer is a short single-stranded nucleic acid molecule that is selected *in vitro* from a large random sequence library based on its high and specific affinity to a target molecule by a process known as SELEX (systematic evolution of ligands by exponential enrichment).^16^, ^17^ Aptamers are applicable to therapeutics by strong and specific neutralizing activities, and they hold several pharmaceutical advantages compared to antibodies, such as a medium size between antibodies and small molecules, chemical synthesis, production cost, and little antigenicity.^18^ The present study demonstrates the therapeutic potential of HA28 in acute cardiac failure.

## Results

**Figure 1:**
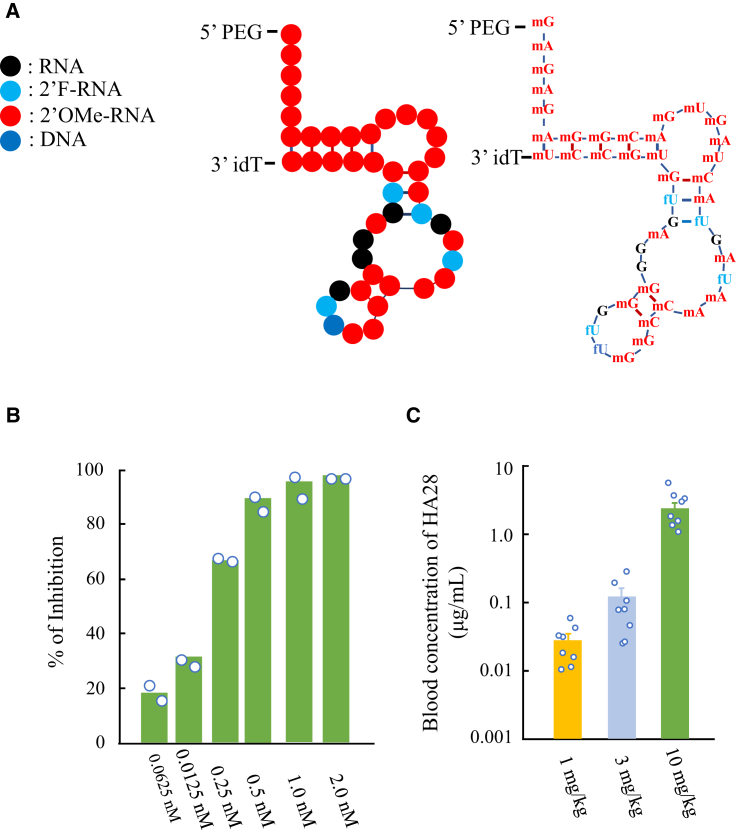
Characteristics of HA28 (A) Structure of HA28. (B) Inhibitory efficacy of HA28 on purified hamster chymase. Each value is the mean of two measurements in the same concentration. (C) Blood concentration 24 hr after final subcutaneous injection of HA28 in the hamster MI model. Each group contained 8 hamsters. HA28 treatment was started 1 day before the induction of MI and continued for 3 days.

The newly synthesized anti-chymase aptamer, referred to as HA28, is 43 nt in length and contains ribose 2′-O-methyl modifications at 33 positions, and 2′-fluoro modifications at five positions, and leaving five 2′ positions unmodified (Figure 1A). (We assumed that 2′-OH groups of these unmodified positions might be directly involved in the interaction with the surface amino acids of chymase because any modifications tested were harmful to the inhibitory activity of HA28). O-methyl or fluorine modifications at ribose 2′ positions and an inverted deoxythymidine (idT) conjugation at the 3′ terminus enabled HA28 tolerant to ribonuclease breakdown. In fact, the half-life (t_1/2_) of HA28 in serum was 20.4 hr. To prolong the half-life of the aptamer by reducing renal elimination during blood circulation, polyethylene glycol (PEG) was added to the 5′ terminus. The molecular weight of HA28 is about 54,900, consisting of 14,900 Da oligonucleotides and 40,000 Da PEG.

To determine the inhibitory efficacy of HA28 on chymase activity, hamster chymase was purified from their cheek pouches, and the 50% inhibitory concentration (IC_50_) of HA28 was determined. As shown in Figure 1B, chymase activity was inhibited at very low HA28 concentrations (0.0625 nM), with a maximal inhibitory effect at 2 nM. The IC_50_ was calculated as 0.17 nM in this study. Figure 1C shows *in vivo* absorption experiments performed in the hamster acute HF models after ligation of the LCA. In these experiments, HA28 at a dosage of 1, 3, or 10 mg/kg was subcutaneously injected 1 day before the induction of MI once a day for 3 days. Collection of blood was performed 24 hr after the final subcutaneous injection of HA28. As shown in Figure 1C, a dose-dependent increase in the HA28 level in blood was observed.

**Figure 2:**
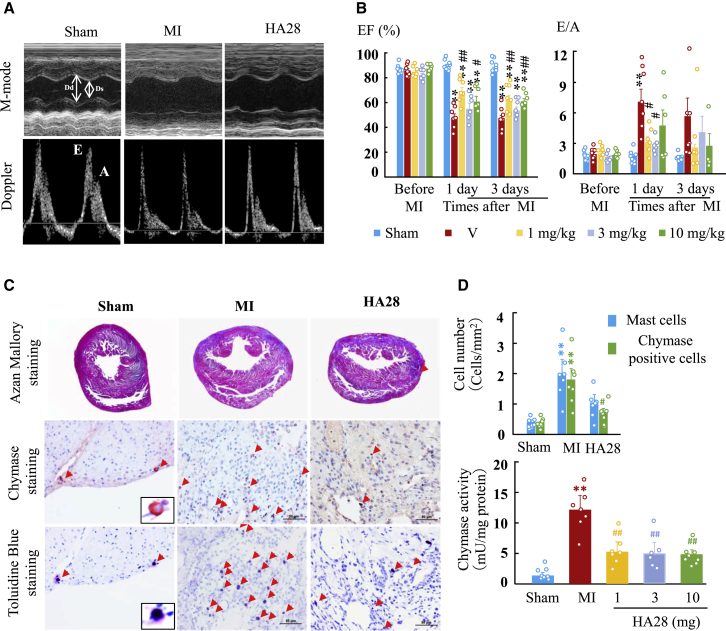
Cardiac Functional and Histological Examinations (A) Representative M-mode echocardiograms and Doppler spectra of mitral inflow in the sham-operated group (sham) and MI hamsters treated with vehicle (MI) or HA28 3 days after the operation. (B) Effects of HA28 on the LV ejection fraction (EF) and the ratio of the early (E) to late (A) ventricular filling velocities (E:A). HA28 at a dosage of 1, 3, or 10 mg/kg was subcutaneously injected 1 day before surgery and continued for 3 days thereafter. (C) Representative azan Mallory staining, toluidine blue staining, and chymase immunohistochemical staining of cardiac sections obtained 3 days after surgery. (D) Effects of HA28 on chymase-positive mast cells and cardiac chymase activities 3 days after MI. HA28 treatment was started 1 day before the induction of MI and continued for 3 days. Dd, left ventricular dimension end diastole; Ds, left ventricular dimension end systole; E, E-wave velocity; A, A-wave velocity. **p < 0.01 versus sham; #p < 0.05, ##p < 0.01 versus vehicle. Each group contained 6–8 animals. Significant differences among the mean values of multiple groups were evaluated with one-way ANOVA followed by post hoc analysis (Fisher’s test).

Before sacrifice of the above animals, the post-MI changes in cardiac function as well as the dose-dependent effects of HA28 on cardiac function in these acute HF models were measured with echocardiography (Figures 2A and 2B). As shown in Figure 2B, the left ventricular ejection fraction (LVEF) and the ratio of the early to late ventricular filling velocities (E:A) were not significantly different among these five experimental groups before MI. However, a significant decrease in LVEF and increase in E:A at the 3-day observation period were very evident after the induction of MI. Compared with MI hamsters treated with vehicle, 1 mg/kg HA28 treatment starting 1 day before surgery improved these cardiac functions significantly. Increasing dosages of HA28 (3 and 10 mg/kg) in these models did not further improve the cardiac function, because we did not find any statistically significant differences among the three HA28-treated groups concerning post-MI cardiac functions.

To determine the infarct size after LCA ligation, azan Mallory staining was performed on cardiac sections obtained from sham-operated hamsters and MI hamsters treated with vehicle or different dosages of HA28. In the vehicle-treated MI group, we found that the infarct size in one hamster was less than 15%, and, therefore, that animal was excluded from the present study. In the 1 mg/kg- and 3 mg/kg-treated MI groups, one and two hamsters, respectively, showed an infarct size below 15%, and they were also excluded from the present study. The infarct sizes calculated in the vehicle-treated and 1-, 3-, and 10-mg/kg-treated groups were 32% ± 3% (n = 7), 37% ± 4% (n = 7), 38% ± 7% (n = 6), and 39% ± 4% (n = 8), respectively.

As shown in Figure 2C, LCA ligation in hamsters induced transmural MI and an MI area that was mainly localized in the left ventricular (LV) free wall. Fibrillar collagen was deposited in the necrotic ventricular wall to replace the lost myocytes, indicating that healing processes had begun in the infarcted LV free wall before this time point. Immunohistochemical studies with an anti-hamster chymase antibody were performed to localize the chymase protein. As shown in Figure 2D, few chymase-positive cells were present in the non-infarcted heart from sham-operated hamsters. However, a marked increase in the numbers of chymase-positive cells was found in the infarcted heart with vehicle treatment. Consecutive sections processed with toluidine blue staining revealed that mast cells were the main source of chymase. The numbers of both chymase-positive cells and mast cells in the infarcted LV were significantly higher than those in the sham-operated group 3 days after the operation (Figure 2D). Chymase-positive cells and mast cells tended to decrease after 3 days in the MI hamsters treated with 1 mg/kg HA28 (Figure 2D).

In parallel with the expression of chymase-positive cells, chymase activity in vehicle-treated infarcted hearts was significantly higher than in sham-operated non-infarcted hearts. On the other hand, equal suppression of cardiac chymase activity was observed in all three HA28-treated groups (1, 3, and 10 mg/kg) (Figure 2D). As mentioned above, 1 mg/kg HA28 treatment provided the maximum improvement in cardiac function, and, given the equal suppression of cardiac chymase activity with the dosage of 1 mg/kg, this dosage was chosen for the following survival-related experiments. On the other hand, we changed the timing of HA28 administration from before MI treatment to after MI treatment, starting 1 day after MI, because the time of cardiac attack is usually unpredictable.

**Figure 3:**
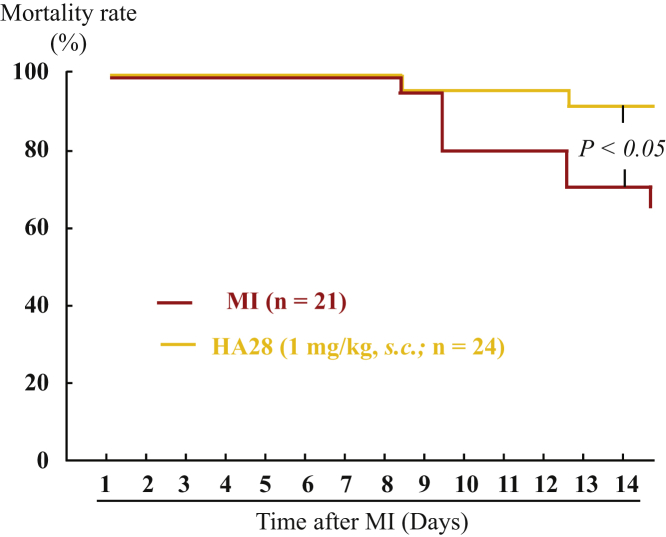
Cumulative Mortality Rates of MI Hamsters Treated with Vehicle (n = 21) or HA28 (n = 24) HA28 at the dosage of 1 mg/kg was subcutaneously injected 1 day after LCA ligation once a day and continued for 14 days. The survival curves of individual groups were compared with the log-rank test.

Figure 3 shows survival curves for the vehicle- and HA28-treated groups during the 14-day observation period after MI. During this observation period, none of the sham-operated hamsters died (data not shown). However, deaths in the vehicle-treated group appeared from day 8 after ligation of the LCA and gradually increased thereafter, with the mortality rate reaching about 33%. HA28 treatment reduced the 14-day mortality rate significantly compared with vehicle-treated hamsters (8%; p < 0.05, vehicle versus HA28), indicating that the inhibition of chymase activity by HA28, even 1 day after MI, improved the outcome following MI.

**Figure 4:**
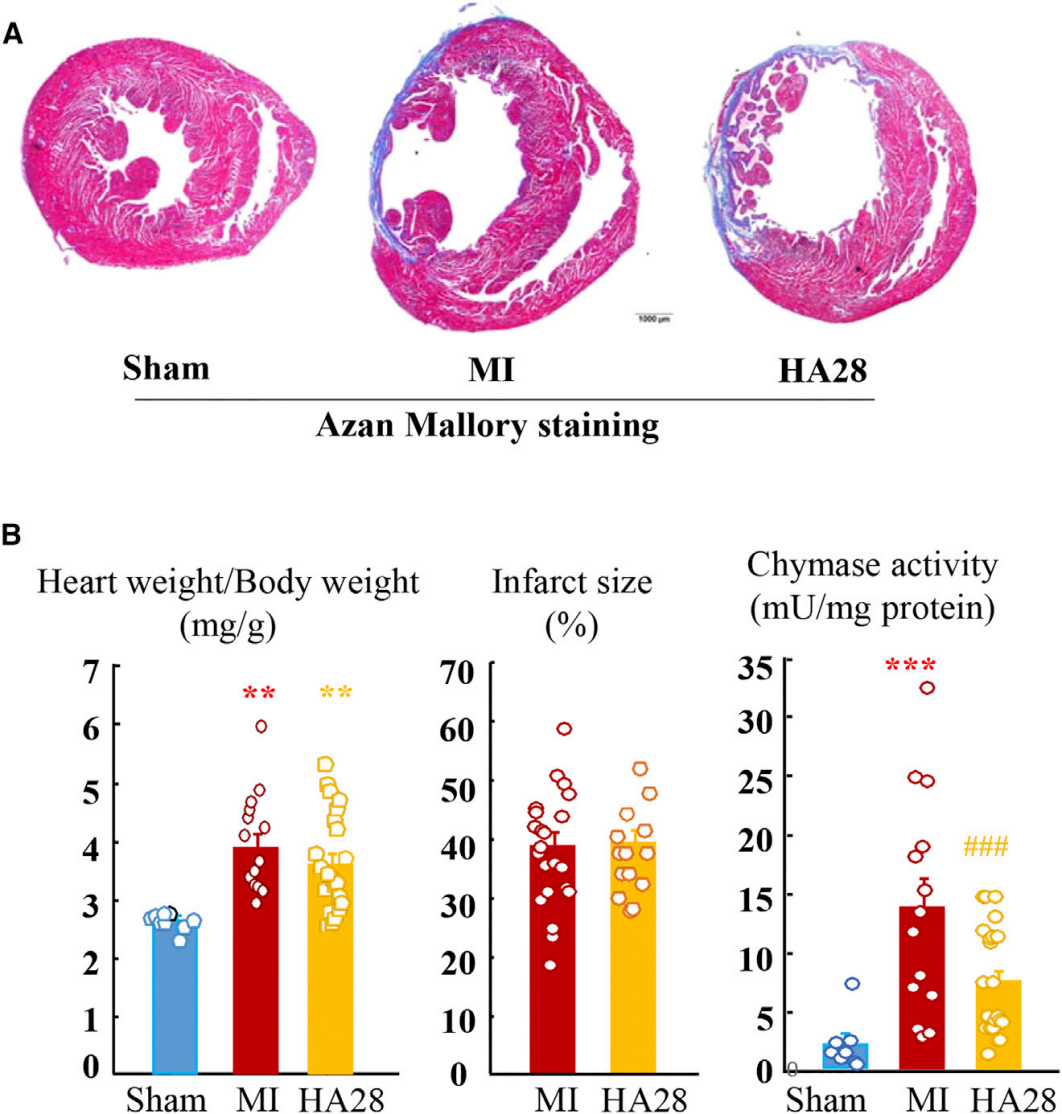
Effect of HA28 on Cardiac Activity, Infarct Size, and Ratio of Heart Weight to Body Weight 14 Days after Surgery HA28 at the dosage of 1 mg/kg was subcutaneously injected 1 day after LCA ligation once a day and continued for 14 days. (A) Representative azan Mallory staining from the sham-operated group and MI groups treated with vehicle (MI) or HA28. (B) Ratio of the whole-heart weight to body weight, infarct size, and cardiac chymase activity in the sham-operated group (n = 8) and MI hamsters treated with vehicle (V, n = 14) or HA28 (n = 22). **p < 0.01, ***p < 0.001 versus sham; ###p < 0.001 versus vehicle. Significant differences between the mean values of two groups were evaluated with the Student’s t test for unpaired data. Significant differences among the mean values of multiple groups were evaluated with one-way ANOVA followed by post hoc analysis (Fisher’s test).

Figure 4A shows representative azan Mallory staining of sham-operated and MI hamsters treated with vehicle or HA28. Compared with the 3-day MI model, the infarcted ventricular wall was thinner, and collagen deposition was more evident. In the vehicle-treated and HA28-treated groups, five and two hamsters, respectively, were excluded from this study because the infarct size was below 15%. No significant differences in the infarct sizes were found between MI hamsters treated with vehicle and HA28. The ratio of heart weight to body weight was increased significantly after MI, and this cardiac hypertrophic response was not affected by treatment with HA28 (Figure 4B). Compared with sham-operated hamsters, cardiac chymase activity was still higher at 14 days after MI. Similar to prophylactic HA28 treatment in the acute HF model, cardiac chymase activation was also inhibited significantly when HA28 treatment was initiated 1 day after MI (Figure 4B).

**Figure 5:**
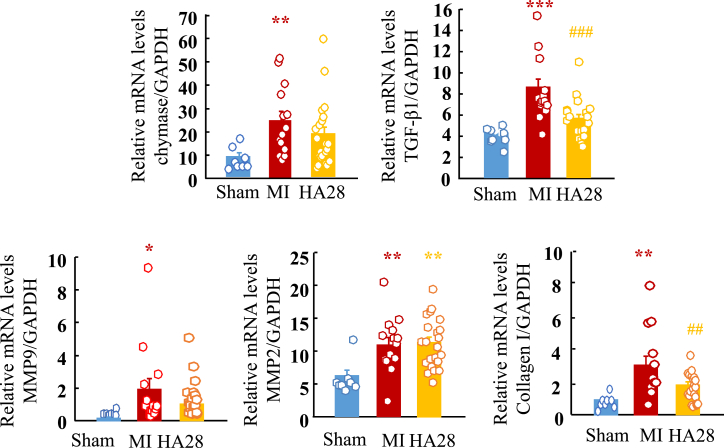
Relative Cardiac mRNA Levels of Chymase, TGF-β1, MMP9, MMP2, and Collagen I in the Sham-Operated Group (Sham) and MI Groups Treated with Vehicle (MI) or HA28 14 Days after Surgery HA28 at the dosage of 1 mg/kg was subcutaneously injected 1 day after LCA ligation once a day and continued for 14 days. *p < 0.05, **p < 0.01, ***p < 0.001 versus sham; ##p < 0.01, ###p < 0.001 versus vehicle.

In the present study, in addition to the cardiac chymase mRNA expression level, we also examined gene expression of several post-MI cardiac remodeling-related factors, such as TGF-β1, MMP9, and collagen I, by using real-time PCR. As shown in Figure 5, compared with sham-operated hearts, mRNA levels of these factors were significantly upregulated after MI. Expression was suppressed to some extent by treatment with HA28, except for MMP2.

**Figure 6:**
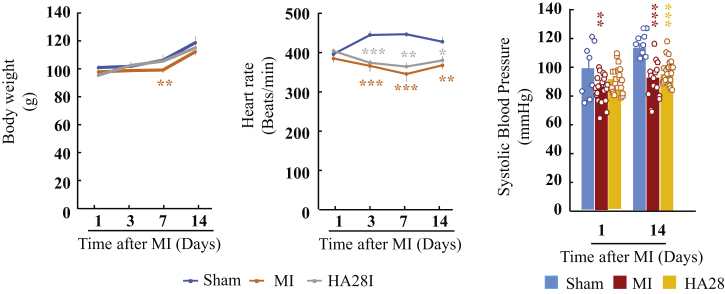
Effect of HA28 on Body Weight, Heart Rate, and Systolic Blood Pressure 14 Days after Surgery HA28 at the dosage of 1 mg/kg was subcutaneously injected 1 day after LCA ligation once a day and continued for 14 days.

Figure 6 shows the effects of HA28 on body weight, heart rate, and systolic blood pressure 14 days after surgery. No obvious differences in body weight were found among the sham-operated, the vehicle-treated, and the HA28-treated groups during 14-day observation periods after surgery. Compared with the sham-operated group, heart rates and systolic blood pressure in both the vehicle-treated and HA28-treated groups were significantly lower.

**Figure 7:**
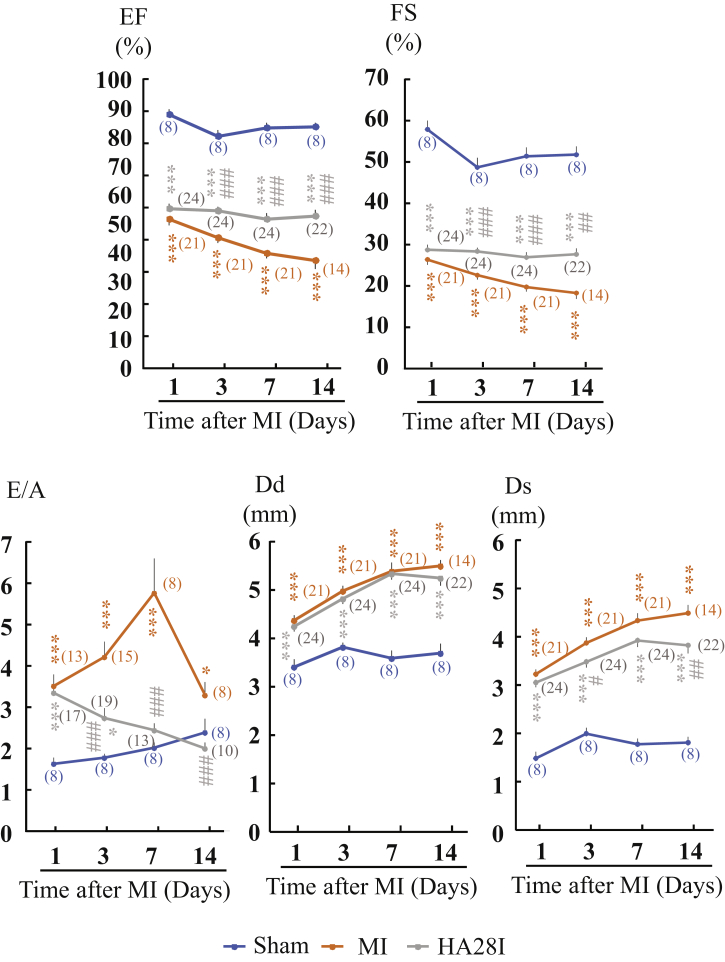
Changes in Echocardiographic Parameters in Sham-Operated Hamsters and MI Hamsters Treated with Vehicle (MI) or HA28 Numerals in parentheses indicate examined numbers of animals. EF, ejection fraction; FS, fractional shortening; Dd, left ventricular dimension end diastole; Ds, left ventricular dimension end systole; E:A, the ratio of the early (E) to late (A) ventricular filling velocities; E, E-wave velocity; A, A-wave velocity. *p < 0.05, **p < 0.01, ***p < 0.001 versus sham; #p < 0.05, ##p < 0.01, ###p < 0.001 versus vehicle.

In these experiments, the chronological effects of HA28 on post-MI cardiac functions during the 14-day observation period were also examined. As shown in Figure 7, echocardiographic parameters such as EF and fractional shortening (FS), which represent cardiac systolic function, were all decreased significantly 1 day after MI. At this time point, we divided the MI hamsters into vehicle-treated and HA28-treated groups according to the evaluation of the EF and FS. In the vehicle-treated group, the EF and FS gradually decreased during the 14-day observation period. However, HA28 treatment prevented the decrease in the EF and FS until 14 days after MI. E:A was also increased significantly after MI in the vehicle-treated hamsters, and these values were reduced significantly in the HA28-treated hamsters at 3, 7, and 14 days after MI, indicating that the inhibition of cardiac chymase could also improve cardiac diastolic function. LV dimension end diastole (Dd) and dimension end systole (Ds) both increased significantly from 1 day after MI. Although HA28 treatment did not affect Dd, it reduced Ds significantly, indicating that improvement in the EF and FS mainly arises from the increase in cardiac contractility after treatment with HA28.

In the HA28-treated MI group, the mean value of the blood concentration of HA28 was 37.95 ng/mL.

## Discussion

The concept of using single-stranded nucleic acids (aptamers) as affinity molecules for protein or compound binding was initially described in 1990.^16^, ^17^ The concept is based on the ability of short oligonucleotides to fold, in the presence of a target, into unique three-dimensional (3D) structures that bind the target with high affinity and specificity.^18^ In the case of the anti-chymase-specific RNA aptamer (HA28), this specific binding should allow the aptamer to hamper the active site of the chymase, by direct blocking or indirect conformational change, and disrupt enzymatic cleavage of target substrates of chymase. Because native RNA is usually degraded quickly *in vivo*, modification of RNA aptamers is necessary to protect them.

In this study, we added O-methyl or fluorine groups to the majority of ribose 2′ positions to protect them from endonucleases. idT was also added to the 3′ terminus of the oligonucleotide to provide resistance to exonucleases. To decrease elimination from kidney, PEG was also added to the 5′ terminus of this aptamer. These modifications and conjugations were incorporated in the oligonucleotide not to affect the binding affinity or inhibitory effect of HA28 on chymase, as shown by our observation that HA28 at a very low concentration inhibited hamster purified chymase activity (Figure 1B).

HA28 is a large molecule. According to the theory of traditional pharmacokinetics, such large molecules are usually unable to penetrate cell membranes, and their absorption and distribution as well as excretion may not occur as desired. However, as shown in Figure 1C, HA28 was detectable in blood after subcutaneous injection, even at a dosage of 1 mg/kg. Surprisingly, the blood concentration remained above 10 ng/mL 24 hr after the final injection of 1 mg/kg HA28 in MI hamsters. Moreover, the chymase activity in cardiac tissues was also inhibited by treatment with 1 mg/kg HA28 (Figures 2D and 4B), indicating that this macromolecule was not only absorbed from locally injected subcutaneous tissues but also was distributed to infarcted heart tissues. HA28 seems to be quite resistant to both RNA exonucleases and endonucleases, because a high blood concentration of HA28 remained even 24 hr after the final injection of 1 mg/kg HA28. Thus, we found that HA28 as a new chymase inhibitor met the requirements for *in vivo* treatment, allowing us to test the effects of this aptamer on cardiac function and survival in the hamster MI model.

In this study, cardiac chymase activity was increased significantly 3 and 14 days after MI (Figures 2D and 4B). Immunohistochemical staining with an anti-chymase antibody showed a significant increase in the numbers of chymase-positive cells in the infarcted hearts, suggesting that an increase in chymase activity is the result of an accumulation of chymase-containing cells in the infarcted heart. Identification of mast cells with toluidine blue staining in serial sections adjacent to chymase-stained sections indicated that mast cells were the main source of chymase (Figure 2C).

In association with the inhibition of post-MI cardiac chymase activity, significant improvements in cardiac functions and the survival rate were observed following treatment with 1 mg/kg HA28 starting 1 day after MI. The decrease in cardiac chymase activity following HA28 treatment may also have resulted from a decrease in the number of mast cells and chymase mRNA expression (Figures 2D, 4B, and 5). The reason why chymase inhibition can reduce the number of mast cells may be explained as follows. Chymase can cleave stem cell factor (SCF) to yield a bioactive soluble product,^19^ and thus it can regulate the number of mast cells in local tissues. SCF is the ligand for Kit, the protein product of the c-*Kit* protooncogene, and c-Kit stimulates the development and differentiated functions of mast cells. These findings indicated that the activation of cardiac chymase following MI plays an important role in post-MI pathology.

We next asked why the suppression of cardiac chymase activation is favorable after MI. Although the present study cannot perfectly address this question, some possible beneficial mechanisms of chymase inhibition were suggested by the results of our study.

In the acute phase of MI, a radical increase in catecholamines and Ang II in blood may be provoked through a vicious circle through arterial baroreflex-mediated activation of the sympathetic nervous system and release of renin from the kidney. The increase in adrenaline facilitates renin release from kidney, and, as a result, both Ang II and aldosterone increase after MI. On the other hand, increased Ang II through the stimulation of AT_1_ receptors present on sympathetic nerve terminals increases noradrenaline release,^20^ resulting in the vicious circle after MI. Therefore, the inhibition of chymase during the acute phase of MI, at least the chymase-dependent Ang II generation that is responsible for this vicious circle, may partially improve cardiac preload and afterload.

In this study, increases in the EF and FS in HA28-treated MI hamsters may reflect an improvement in cardiac afterload following chymase inhibition (Figure 2B). Another benefit of chymase inhibition may be a reduction in the occurrence of arrhythmia after MI. Many types of arrhythmias are triggered frequently by the excessive stimulation of β1 adrenergic receptors.^21^ Moreover, the arrhythmogenic effects of Ang II during cardiac ischemia have been shown in previous studies.^22^ In addition to these above observations, we have found that chymase inhibition can provide an antiarrhythmic effect on a canine LCA ligation model.^23^ Taken together, our observations indicate the beneficial effects of chymase inhibition during the acute phase of MI.

In the chronic phase of MI, to keep CO as close as possible to the normal condition, remaining surviving cardiomyocytes are needed to become hypertrophic myocytes and support MI survivors for their lifetime. Once the heart has lost its cardiomyocytes after MI, new cardiomyocytes are usually unable to regenerate. Therefore, in post-MI hearts, an increase in the size of individual remaining cardiomyocytes may be the best way to compensate for the decreased CO. Noradrenaline, adrenaline,^9^ TGF-β1,^24^ and Ang II^8^ play very important roles in these compensatory processes, and these molecules may be necessary during the acute phase of MI. However, persistent activation of these neurohumoral systems sometimes incurs unfavorable results. For example, in addition to the hypertrophic process, the deposition of collagen fibers throughout the interstitium of cardiomyocytes will progress gradually following the chronic phase of MI, and, finally, a severe decrease in cardiac function induced by increased stiffness as well as disruption of the cardiac impulse conductive pathway may occur. Ang II,^25^ TGF-β1,^26^ and MMPs^27^ are believed to play pivotal roles in the pathogenesis of such remodeling. The therapeutic effects of a chymase inhibitor on the chronic state of MI may also be expected, due to its role in the activation of these remodeling factors. Although we did not examine the chronic phase after MI in this study, as shown in Figure 5, the expression of TGF-β1, MMP9, and collagen was significantly increased in cardiac tissues 14 days after MI, indicating that cardiac fibrotic procedures had already been initiated at this time point. We found that the expression of these mRNAs was partially suppressed by treatment with HA28.

The possible favorable effects of chymase inhibition after MI from animal experiments were reported as early as 2001,^28^, ^29^, ^30^, ^31^ and, based on these accumulated data, a clinical phase II trial (ClinicalTrials.gov: NCT02976467) aimed at confirming the effects of a chymase inhibitor on cardiac function in patients with acute MI is now ongoing in Europe. Whether a chymase inhibitor as a new therapy can also exert beneficial effects on post-MI pathophysiology in patients is now attracting much attention among cardiologists.

In conclusion, the present study provides the first evidence that a single-stranded RNA aptamer that functions as a chymase-specific inhibitor is very effective for the treatment of acute HF caused by MI. In addition, the inhibition of cardiac chymase activity with HA28 not only significantly improved post-MI cardiac function but also resulted in reduced mortality during the acute phase of MI, indicating again that chymase may be a new therapeutic target for post-MI pathophysiology.

## Materials and Methods

### Screening of an Anti-hamster Chymase RNA Aptamer

Selection and modification of an anti-hamster chymase RNA aptamer were carried out according to the SELEX methods described by Oguro et al.^32^ Extensive selection or amplification was performed in 52 independent SELEX conditions with different libraries, modified nucleotides, and different primer sets to isolated aptamers that selectively bind to hamster chymase. The resulting aptamer candidates were examined for the binding affinity using a surface plasmon resonance measurement^32^ and the enzyme assay, as described by Takai et al.^10^ The candidate aptamers were subjected to size shortening and optimization by ribose 2′ modifications, and they were further modified at 3′ and 5′ termini with idT and PEG, respectively, giving rise to the final aptamer HA28. HA28 exhibited specific and strong affinity to hamster chymase. Then the IC_50_ of HA28 was determined with purified hamster chymase, according to the methods described by Takai et al.^10^ To confirm the bioavailability of HA28, we measured the blood concentration of HA28 in hamster 3-day MI models. HA28 at dosages of 1, 3, and 10 mg/kg was subcutaneously injected beginning 1 day before the induction of MI once a day for 3 days. The detailed numbers of animals in each group are reported below. Blood was collected 24 hr after the final injection. The HA28 concentration in plasma was determined by using an enzyme-linked oligonucleotide sorbent assay.^33^

### Establishment of Hamster LCA Ligation and Sham-Operated Models

Male Syrian hamsters (SLC, Shizuoka, Japan) aged 6 weeks and weighing 90–110 g were used. The experimental procedures for animals were conducted in accordance with the guidelines of Osaka Medical College for medical experiments, and they were approved by the ethics committee (29004). The hamsters were fed regular hamster chow; had free access to tap water; and were housed in a temperature-, humidity-, and light-controlled room. MI was produced in hamsters using previously described techniques.^28^ In brief, a left-sided thoracotomy was performed under anesthesia. After opening the chest, the LCA was ligated near its origin, and then the thoracotomy site was closed in layers. Some hamsters underwent a sham ligation; a similar surgical procedure was performed, except that the suture was not tightened around the coronary artery (sham).

### Grouping

To examine the *in vivo* inhibitory efficacy of HA28 on chymase activity and to measure the blood concentration of HA28, the LCA-ligated and sham-operated animals were divided into five groups: sham-operated (n = 8), vehicle-treated (n = 8), 1 mg/kg HA28-treated (n = 8), 3 mg/kg HA28-treated (n = 8), and 10 mg/kg HA28-treated (n = 8) groups. In these animals, the vehicle-treated group received vehicle treatment, and the HA28-treated groups received subcutaneous injection of the anti-chymase aptamer HA28 starting 1 day before surgery and continuing until 3 days after MI. At 4 days after surgery, blood samples were collected to measure the blood concentration level of HA28. Thereafter, heart samples were harvested for later biochemical and histological examinations.

To examine the effects of chymase inhibition by HA28 on post-MI cardiac function and the survival rate, the following protocols were employed. Eight hamsters underwent sham operation to create the sham group; 52 hamsters underwent LCA ligation to prepare MI model hamsters. At 1 day after MI, these MI model hamsters were divided into the vehicle-treated and HA28-treated groups after the evaluation of LVEF with echocardiography. The vehicle-treated group received a subcutaneous injection of saline (0.2 mL/100 g) once a day. The HA28-treated group received a subcutaneous injection of HA28 at a dosage of 1 mg/kg/day.

During the 14-day observation period, echocardiographic parameters, such as EF and the ratio of E-wave velocity to A-wave velocity (E:A ratio), were calculated in each group at 3, 7, and 14 days after MI. During the treatment period, cages were inspected daily for animals that had died. At 14 days after MI, blood samples were collected for later measurement of the blood concentration of HA28. Meanwhile, both sham-operated and infarcted hearts were harvested after the collection of blood samples, and each heart was divided into three parts from the cardiac basalis to the apex. Among these sectioned parts of the heart, the middle part was used to determine the infarcted area, and the remaining two parts were used for the biochemical parameter measurements.

### Blood Pressure Measurement and Echocardiographic Study

Arterial systolic blood pressure was measured (non-invasive NP-NIBP MONITOR, MK-2000ST, Muromachi Kikai, Tokyo, Japan) under 2% isoflurane inhalation with an anesthesia machine (NARCOBIT-E (II), KN-1071, Natsume Seisakusho, Tokyo, Japan).

Echocardiographic studies were performed under isoflurane anesthesia using an echocardiographic system (Vevo 1100 Imaging System, Transducer: MS250, FUJIFILM VisualSonics, Toronto, ON, Canada), according to previously described methods.^29^

### Histological Studies

The infarct size was determined on a section stained with azan Mallory by using a computerized morphometry system (NIS-Elements Documentation, version (v.) 3.07, Nikon, Tokyo, Japan). The infarct size was expressed as a percentage of the infarcted length to the total LV circumference. Mean infarct sizes of less than 15% were excluded from the analysis.

To identify the distribution of mast cells and chymase-positive cells, toluidine blue staining and immunohistochemical staining were performed on two serial sections. Detailed methods have been described elsewhere.^28^ In brief, toluidine blue was dissolved to 0.5% in distilled water and the pH was adjusted to 4.8. Deparaffinized sections were immersed in 0.5% toluidine blue solution for about 15 min, fractionated with 0.5% glacial acetic acid solution, and mounted after drying. Chymase immunohistochemical staining was performed with an anti-hamster chymase antibody (raised in rabbit by immunization with SPYVPWINIVIKASS, which is the sequence of the C-terminal amino acid residues from positions 212 to 226 of hamster chymase, kindly given to us by Otsuka Pharmaceutical, Tokushima, Japan). Deparaffinized sections were incubated with anti-hamster chymase antibody (1:400) for 1 hr at room temperature, followed by reaction with components from a labeled streptavidin-biotin peroxidase kit (Dako LSAB kit, Dako, Carpinteria, CA, USA) that included 3-amino-9-ethylcarbazole color development. The sections were lightly counterstained with hematoxylin. The numbers of mast cells and chymase-positive cells within the LV area were quantified using a computerized morphometry system and expressed as the absolute number of stained cells per square millimeter of LV.

### Measurement of Biochemical Parameters

Chymase activities were measured according to previously described methods.^10^ In brief, cardiac tissues were homogenized in 20 mM Na-phosphate buffer (pH 7.4), centrifuged at 15,000 × *g* for 30 min, and the supernatant was discarded. The remaining pellets were resuspended and rehomogenized in 10 mM Na-phosphate buffer (pH 7.4) containing 2 M KCl and 0.1% Nonidet P-40. The homogenate was recentrifuged at 15,000 × *g* for 30 min, and the supernatant was used for measurements of chymase activity. Chymase activity in the cardiac extract was measured by incubating the tissue extracts with 1 mM Ang I (Peptide Institute, Osaka, Japan) as the substrate.

Real-time PCR was used to examine the expression of chymase, TGF-β1, MMP2, MMP9, and collagen I in cardiac tissues, with the previously described methods.^34^ In brief, after the extraction of cardiac total RNA with TRIzol reagent (Life Technologies, Rockville, MD, USA), the extracted RNA (1 μg) was reverse transcribed into cDNA with SuperScript VILO (Invitrogen, Carlsbad, CA, USA). Then, mRNA levels were measured with real-time PCR on a Stratagene Mx3000P (Agilent Technologies, San Francisco, CA, USA) using TaqMan fluorogenic probes. The mRNA levels of chymase, TGF-β1, MMP2, MMP9, and collagen I were normalized to that of glyceraldehyde-3-phosphate dehydrogenase (GAPDH).

### Statistical Analysis

All numerical data shown in the text are expressed as the mean ± SEM. Significant differences between the mean values of two groups were evaluated with the Student’s t test for unpaired data. Significant differences among the mean values of multiple groups were evaluated with one-way ANOVA followed by post hoc analysis (Fisher’s test). Survival data are presented as Kaplan-Meier curves. The survival curves of individual groups were compared with the log-rank test. p < 0.05 was considered statistically significant.

## Author Contributions

D.J. and Y. Nakamura conceived and managed the study and wrote the manuscript. S.T. measured the chymase activity and its gene expression. Y. Nonaka measured the blood concentration of HA28. S.Y., M.F., and Y. Nakamura contributed to the construction of HA28.

## Conflicts of Interest

Except D.J., S.T., and S.Y., all authors are employees of and hold equity in RIBOMIC Inc. The remaining authors declare no competing financial interests.
